# Therapeutic Use of Native and Recombinant Enteroviruses

**DOI:** 10.3390/v8030057

**Published:** 2016-02-23

**Authors:** Jani Ylä-Pelto, Lav Tripathi, Petri Susi

**Affiliations:** 1 Department of Virology, University of Turku, Kiinamyllynkatu 13, 20520 Turku, Finland; jani.ylapelto@gmail.com (J.Y.-P.); lav.tripathi@utu.fi (L.T.); 2 Biomaterials and Diagnostics Group, Turku University of Applied Sciences, 20520 Turku, Finland

**Keywords:** CAVATAK™, coxsackievirus A21, enterovirus, poliovirus, IRES, PVS-RIPO, virotherapy

## Abstract

Research on human enteroviruses has resulted in the identification of more than 100 enterovirus types, which use more than 10 protein receptors and/or attachment factors required in cell binding and initiation of the replication cycle. Many of these “viral” receptors are overexpressed in cancer cells. Receptor binding and the ability to replicate in specific target cells define the tropism and pathogenesis of enterovirus types, because cellular infection often results in cytolytic response, *i.e.*, disruption of the cells. Viral tropism and cytolytic properties thus make native enteroviruses prime candidates for oncolytic virotherapy. Copy DNA cloning and modification of enterovirus genomes have resulted in the generation of enterovirus vectors with properties that are useful in therapy or in vaccine trials where foreign antigenic epitopes are expressed from or on the surface of the vector virus. The small genome size and compact particle structure, however, set limits to enterovirus genome modifications. This review focuses on the therapeutic use of native and recombinant enteroviruses and the methods that have been applied to modify enterovirus genomes for therapy.

## 1. Introduction

**Figure 1:**
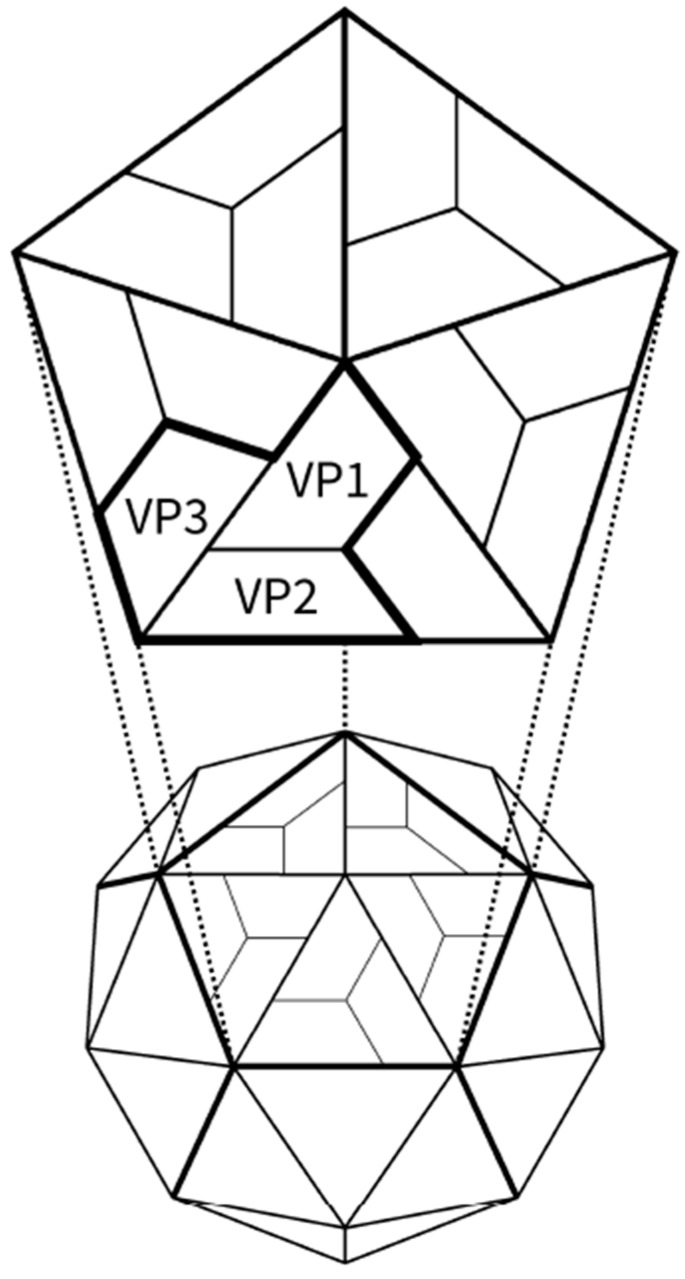
Schematic presentation of enterovirus structure. Icosahedral capsid of enteroviruses is composed of 12 pentameric units. The pentamer contains five protomeric subunits around five-fold symmetry axes. Positions of surface-exposed capsid proteins VP1, VP2 and VP3 are shown.

**Figure 2:**
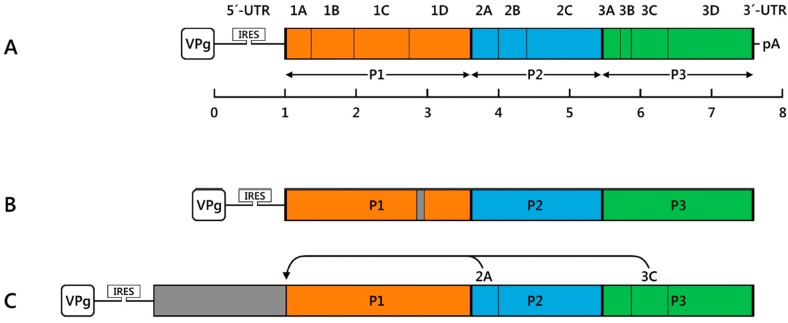

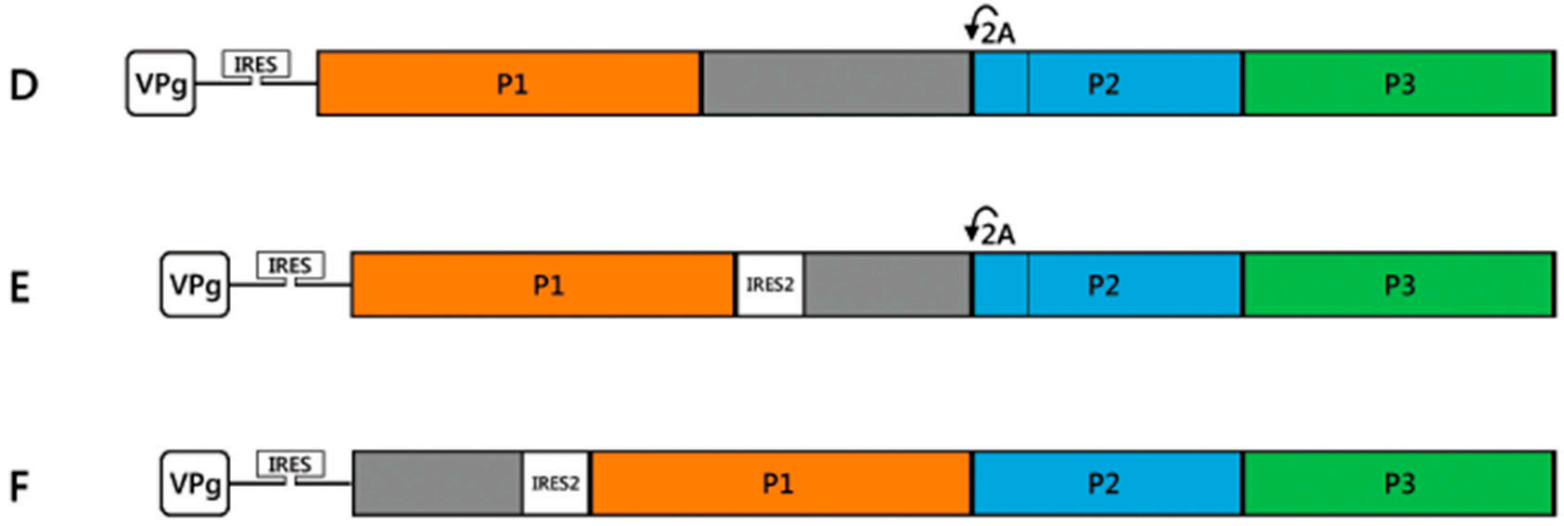
The genetic structure of enterovirus expression vectors. (**A**) Schematic representation of the enterovirus genome (drawn in the scale except for the internal ribosomal entry site (IRES) region and the inserts); the conserved and highly-structured 5′- and 3′-untranslated regions (UTRs) are indicated and the major proteolytic products of the viral polyprotein (P1, P2, and P3) are outlined by colored boxes. The 5′-end of the genome is covalently bound by viral genome-linked protein, VPg and 5′-UTR contains an IRES for cap-independent translation. The 3′-UTR contains a short polyA tail (pA); (**B**) Epitope display vectors contain short foreign inserts to display epitopes (in grey) within the P1 structural protein-encoding region; (**C**,**D**) Protein expression vectors. Foreign sequences fused to the viral ORF at its 5′-end or between the coding regions for P1 and P2. Artificial proteolytic cleavage sites for the viral 2A^pr^° and/or 3C^pr^° proteases (indicated by curved arrows) are used for proteolytic processing of the fusion polyprotein; (**E**,**F**) Dicistronic vectors. Foreign inserts placed under the control of a secondary IRES element (IRES2) (originating from a related picornavirus) between P1 and P2. Alternatively, the upstream cistron is formed by the foreign insert driven by the original IRES, and the viral polyprotein is expressed under the control of the IRES2.

Picornaviruses form one of the largest RNA virus groups. At present, the family *Picornaviridae* contains 26 genera and three proposed genera, which include several important human and animal pathogens causing a range of diseases from infections of the central nervous system to respiratory illnesses [1]. The genus *Enterovirus* includes polio-, rhino- (the common cold virus), coxsackie- and echoviruses, as well as numbered enteroviruses, which account for most of the known picornavirus types (currently more than 270 enterovirus types have been identified). Enterovirus particles are non-enveloped and small in size (28 nm in diameter). Icosahedral capsids are composed of 60 copies of each of the capsid proteins (VP1 to VP4) (Figure 1) that enclose an infectious, positive-sense, single-stranded RNA genome, approximately 7.1–8.9 kb in length (Figure 2A) [1,2]. The RNA genome functions as mRNA, which is encoded into a large polyprotein via the internal ribosome entry site (IRES) translation mechanism. The polyprotein is auto-catalytically cleaved into functional structural and non-structural proteins by viral-encoded proteases, resulting in virus replication and eventually the formation of intact virus particles [1,3,4].

Viral capsid protein(s) contain specific motifs that mediate virus-binding to cell surface receptors to initiate the replication cycle. Enterovirus receptors include poliovirus receptor, Neclin-5 (Necl-5), intracellular adhesion molecule-1 (ICAM-1), coxsackie-adenovirus receptor (CAR), decay accelerating factor (DAF), low density lipoprotein (LDL), SCARB2 and integrin receptors, but for most enteroviruses the receptor is not known because experimental studies have gathered around model enterovirus types. Non-protein factors such as heparan sulphate and sialic acid also mediate enterovirus infection [1,3,5,6]. Importantly, many of the protein receptors are overexpressed in cancer cells, which makes native enteroviruses potential tools for oncolytic virotherapy. In addition, the generation of enteroviral cDNA clones has enabled not only studies of virus replication and the role of viral proteins in it, but also the development and use of modified enteroviruses in gene therapy, or in oncolytic virotherapy. This review focuses on the methods that have been applied to modify enterovirus genomes for therapy. In addition, we will review the use of native and recombinant enteroviruses, especially in oncolytic virotherapy.

## 2. Modification of Enterovirus Genome Is Complicated due to Technical and Space Limitations

There are basically two reasons that have limited the modification of enteroviruses: (1) the lack of feasible methods to obtain viable particles from the viral RNA genome and (2) the structural limitations of the genome and the capsid, which often lead to the instability of recombinant virus particles. Even though the size of the plus-sense RNA genome of enteroviruses is relatively small (7.1–8.9 kb), straightforward genome modification methods—from viral RNA to the recovery of mutated virus particles—have only recently been employed. In the past, cDNA clones of many enteroviruses were generated by step-by-step cloning of compatible restriction enzyme–digested or PCR-amplified fragments into mammalian expression vectors, and by integration of a specific cleavage site to the 3′-end of the viral genome for linearization of the viral vector [7,8,9]. The linearized template was then subjected to the *in vitro* transcription reaction, and the RNA transcripts were transfected into mammalian cells from which infectious enterovirus particles were recovered. Mutagenesis of enterovirus cDNA clones was typically carried out in a time-consuming process including subcloning of the target region into a propagation plasmid, PCR of the target region by mutagenic primers and subcloning of the target back to the viral cDNA backbone using compatible restriction enzyme–cutting sites [8,10,11,12,13]. Interestingly enough, such methods are still being used [12,13] even if high fidelity, long-range PCR enzymes have already been used to amplify long genomic fragments or even full-length genomes in a single step 20 years ago [14,15,16,17]. More recent studies have included long PCR protocols for cDNA cloning [18,19,20,21]. The use of T7-RNA polymerase-driven transcription together with the ribozyme hammerhead sequence enables the efficient generation of progeny virus directly in the target cells [18,22,23], making the overall process short and feasible. Gene synthesis *in vitro* allows the direct generation of (mutated) viral fragments or even full-length viral genomes, alleviating cloning using the viral RNA as a template. Gene synthesis is particularly useful in generating a precise terminal 5′-end to the viral cDNA clone since a lack of it may result in reduced ability to replicate [22]. However, this is only useful if the 5′-end of the template has been verified by the rapid amplification of cDNA ends (RACE) or Next Generation Sequencing (NGS) methods. Most enteroviral full-length sequences deposited in the GenBank have been generated using generic primers, and it is necessary to verify the exact termini before experimentation and vector generation. Anyhow, modern techniques make it possible to generate recombinant viruses within a matter of a few weeks.

Although seemingly easy, the compact size and the structure of the enterovirus genome limit the size of the inserts or generation of the protein fusions when preparing recombinant viruses. The enterovirus genome is composed of a 5′-terminal untranslated region (UTR) followed by the structural proteins (VP4, VP2, VP3 and VP1), non-structural proteins (2A–C and 3A–D), and short UTR at the 3′-terminus (Figure 1A). The enteroviral RNA lacks a 5′-terminal cap structure and therefore relies on translational initiation through internal ribosomal entry [4,24]. The internal ribosomal entry site (IRES), a complex cis-acting genetic element with an extensive secondary structure, mediates cap-independent translation of the viral genome. During the replication cycle a long polyprotein is translated and processed into functional viral proteins by viral proteases (2A^pr^° and 3C^pr^°) [1,4,25]. The structural integrity and polyprotein translation strategy thus define the useful sites for genetic insertion. Earlier studies to identify the optimal size and site for the insert have been important because the site has a significant effect on the stability of the genome. These studies have resulted in identification of several sites, which have been used to generate viable and stable recombinant human enteroviruses. The strategies for vector development using some of the most important enteroviruses will be addressed in the following chapters.

## 3. Recombinant Enterovirus Vectors

Enteroviruses and particularly poliovirus-1 (PV-1) and coxsackieviruses B3 (CV-B3), CV-B4 and CV-A9 have been used to develop viral vectors from both basic research and therapy. They cause both acute and chronic diseases, which is why they are widely used model viruses. There are specific reasons why enteroviruses are optimal viruses for vector use: They do not integrate into the human genome, they replicate in non-dividing cells (to some extent), they possess cell tropism, and there are infectious cDNA clones and mouse models available for virus studies and development of a gene therapy vector. Due to the global poliovirus eradication campaign, it is likely that the other enteroviruses besides PV will attract more attention as gene vectors in the near future. Various strategies have been employed to express foreign genes from enterovirus vectors (Figure 2). The vector design is based on the known principles governing picornavirus gene expression: Short inserts encoding antigenic epitopes have been placed within the capsid protein [26,27,28,29]. Viable recombinants can also be generated by placing inserts either in front of the VP4 gene or cloning the insert with flanking protease cleavage sites to the 3′-end of the VP1 structural protein gene or between the non-structural genes 2A and 3C (Figure 1C,D) [18,30,31,32,33]. Finally, functional recombinants have been obtained by using two internal IRES sites in the same genome followed by the insert (Figure 2E,F). Although each strategy may have its limitations, the aim ultimately defines which strategy is to be used. For example, if recombinant virus is used directly in target cells, the stability of the virus is not an issue. Instead, if the virus is used for cell targeting, which includes several replication cycles, the vector must be very stable. Use in gene or cancer therapy is often accompanied by the expression of therapeutic protein from the genome, and in that case, the size of the insert may become the limiting factor. In the following chapters, we will highlight some of the work related to the generation and use of enteroviruses in gene therapy and vaccination (Table 1).

### 3.1. Poliovirus Vectors

Poliovirus is one of the most devastating human pathogens, and it has been intensely studied over the years. PV has been the forerunner in studies where recombinant enteroviruses have been designed for therapeutic use. Several strategies have been employed to engineer poliovirus expression vectors. Small antigenic epitopes have been inserted into VP1 capsid protein between the P1 and P2 or between the P2 and P3 regions, and used in vaccination studies [30,32]. The 5′-terminal IRES sequence has been duplicated (one IRES from PV and the other from a related rhinovirus) to generate a dicistronic poliovirus; polioviral proteins are produced from native IRES, and the second IRES controls the expression of the inserted gene [38]. Replacement of the capsid gene and virus packaging *in trans* using capsid proteins from a helper Vaccinia virus resulting in the generation of viable recombinant viruses has also been feasible [29,37,42,43]. However, this strategy does not work *in vivo* because no progeny virus is produced. The above-mentioned strategies have limitations in insert size, instability of the vector or requirement for a helper virus. More recently, inserts have included flanking artificial protease cleavage sites, which enable stable recombinants with large inserts to be generated [44]. This strategy is based on the auto-catalytic cleavage of the insert cloned into the 5′-terminus preceding the VP4 gene or in the junction between the VP4/2 or VP1/2A genes by two the viral proteases 2A^pr^° and 3C^pr^°/3CD^pr^° [35,44,45,46]. Dobrikova *et al.* [35] studied the tolerance to insert size by replacing the PV-1 (Sabin strain) IRES with rhinovirus 2 (RV-A2) IRES. The loop area IV of RV-A2-IRES was then replaced with inserts of different sizes (102, 282, 402 and 744 nucleotides) followed by the 2A^pr^° cleavage site. Although all recombinant viruses produced progeny viruses, there were differences in the stability of the inserts, and based on this study the insert size was limited to 300 nucleotides in stable recombinants. This indicates that this strategy may not be optimal for heterologous protein expression, e.g., to be used as a vector for vaccination. Anyhow, this strategy with a protease cleavage site was able to preserve the insert better than the previously attempted strategies. In addition, it was also noted that modification of the secondary structure loops of the IRES domain had considerable effect on the virus stability, which is important for the mass-scale production of a vector virus where multiple rounds of passages take place.

Poliovirus has been regarded as a prominent virus vector for the gene therapy of neuron-related diseases in the central nervous system (CNS) because, during multicellular infection, the virus is confined almost exclusively to neurons. To reduce the pathogenicity of the vector, vectors with the capacity to encapsidate RNA replicons *in trans* have been generated. Viral replicons encoding the target gene in place of PV capsid genes are trans-encapsidated by providing the capsid proteins using a recombinant Vaccinia virus, which encodes PV capsid proteins. Since the replicons do not encode PV capsid proteins, they undergo only one round of infection [38]. Intracranial or intraspinal introduction of PV replicons into mouse CNS did not result in any signs of pathogenicity when the luciferase marker protein was transiently expressed [47]. In another study, the PV vector and trans-encapsidation was used to study how mouse interleukin 10 (mIL-10) affects the regeneration of spinal cord injuries [36]. Mice injected in the spinal cord expressed considerable levels of mIL-10 in the brain tissue compared to mice injected in the muscle. As indicated above, this strategy is optimally used in direct inoculation of the virus vector into target tissue, and is therefore suited only for specific therapeutic use.

To alleviate the problems in vector delivery, a replication-competent recombinant virus has also been generated. The PV-1 Mahoney strain was modified and used to express brain-derived neurotrophic factor (BDNF) in mice that express poliovirus receptor CD155 [34]. The insert was cloned to the 5′-end of the genome with an N-terminal artificial 3C^pr^° cleavage site. The expression of BDNF in the neuronal cells was transient and there were no signs of pathogenicity, which were attributed to the lower replication level of the vector [34]. Although the transient expression system may be safer than the use of replication-competent poliovirus vectors, the expression is also less efficient. Anyhow, both strategies employ the natural targeting of PV into neurons in the CNS, which promotes the development of poliovirus-based vectors for neural gene delivery. Currently, one such vector, recombinant oncolytic poliovirus (PVS-RIPO), is in clinical trials for cancer therapy.

### 3.2. Coxsackievirus Vectors (CV-B3, CV-B4 and CV-A9)

While the first poliovirus-derived vector constructs were already generated in the late 1980s, other enteroviruses were used much later, some as recently as 15 years ago [39]. Coxsackievirus B3 (CV-B3) has been the most studied coxsackievirus for gene therapy purposes because of its prevalence and role in myocarditis and pancreatitis [48]. However, due to its pathogenicity, CV-B3 virus must be attenuated before its use as a vector virus. There are several kinds of attenuated CV-B3 strains. The attenuation phenotype is often linked to virus inability to bind to a cellular receptor [33,48]. The strategies used to construct recombinant CV-B3 include insertion of small gene fragments within VP1, a dicistronic system with an extra IRES from a related picornavirus, the trans-encapsidation strategy, and cloning of the target gene between the 5′ non-translated region (5′ UTR) and VP4, or between the VP1 and 2A genes flanked by artificial 2A^pr^°/3C^pr^° cleavage sites. Similarly to the PV vector, the size of epitopes expressed in the VP1 structural protein is limited to nine to 10 amino acids [10,49] which is due to structural constraints. Instead, insertion between structural and non-structural genes allows large insert sizes (up to 750 nucleotides) because the release of the foreign protein does not interfere with the polyprotein processing, which is why most studies based on recombinant CV-B3 have used this method. However, when generating recombinants it is important to use non-identical protease cleavage sites flanking the insert, because in most cases, recombinant viruses are stable up to 10 rounds of passage. This has been shown to increase the stability of the vector [7].

CV-B3 was first used for vaccination experiments by Höfling *et al.* [39] who introduced the Adenovirus 2 (Ad2) L1 hexon antigen between VP1 and 2A^pr^° proteins to test a multivalent vaccine with Ad2 and CV-B3. In HeLa cells, wild-type and chimeric viruses reproduced significantly faster compared to the COS-1, HCAEC and MFHF cell lines. The yield of the recombinant virus was poor due to the delayed polyprotein processing, but the stability of the recombinant was good. Immunized BALB/c mice produced antibodies against both viruses. Chapman *et al.* [7] demonstrated the expression of a functional murine interleukin-4 protein, which induced a humoral immune response against CV-B3 and was stably expressed for 14 days.

CV-B3 replication is affected by the cell cycle, which may affect its usability in gene therapy—to its benefit. Feuer *et al.* [40] used green fluorescent protein (GFP) as a marker and found out that virus replication is dependent on the cell cycle. In cells that were suspended in G0 or G2/M phases, virus production was slowed down considerably and the virus production stayed latent for three days even though virus RNA was present inside the cells. When the cell cycle was allowed to proceed, virus production also continued. Similar results were obtained when confluent, infected cells were wounded *in vitro*. GFP gene expression was only detected on the edges of the wound. The GFP gene was also used in another study to monitor virus replication in non-dividing cardiac muscle cells [9]. The GFP gene was cloned to the 5′-end of the CV-B3 genome. Recombinant virus replicated almost at the same rate in COS-1 cells as in the wild-type CV-B3-H3, but in cardiac muscle cells the expression was weak. However, the lower level of virus replication together with the attenuated phenotype are likely to make the virus less pathogenic, yet allow antigenic epitope or therapeutic protein expression, which is sufficient for the induction of immune response or results in the healing process [33,48,50]. Overall, the data suggest that attenuated CV-B3 may be suitable for vaccination and gene therapy (especially in gene transfer into cardiac cells).

Coxsackievirus B4 (CV-B4) is a close relative to CV-B3, and besides typical enteroviral diseases, it is linked to the development of type 1 diabetes (T1D) [51,52]. Even if CV-B4 has been used only in a few vaccination studies, they indicate its potential as a gene therapy vector; such a vector could be used, for example, in the development of a T1D vaccine. Halim *et al.* [28] studied the expression of ovalbumin in the DE loop, which links amino acid strands D and E, in the CV-B4 VP1 structural protein (amino acids 128–140). This loop has been implicated in having an effect on the virus stability and pathogenicity. They showed that the insert size was limited to 10 amino acids, which is similar to PV and CV-B3. They also constructed a recombinant CV-B4 virus, which expressed three fragments of the human immunodeficiency virus type 1 (HIV-1) p24^gag^ gene cloned into two different sites: in the VP1 gene and between 5′ UTR and VP4. Recombinant virus carrying two fragments of the HIV-1 p24^gag^ gene in the VP1 DE loop was stable for several passages, while recombinants that had the insert between 5′ UTR and VP4 were labile [41].

Coxsackievirus A9 (CV-A9) differs from the other members of the *Enterovirus B* species in that it uses the cell surface integrins as receptors for cellular entry by binding via the RGD motif that resides in the VP1 capsid protein [53,54]. The CV-A9 vector could be useful when targeting cancer cells that overexpress αVβ3 integrin, *i.e.*, in anti-angiogenic therapy [55]. To generate a recombinant virus, the eGFP gene was inserted into the cDNA clone of CV-A9 between the VP1 and 2A genes with flanking protease sites, resulting in a virus vector, which was stable for nine passages. This study also described a method for rapid cloning, mutagenesis and transcription *in vivo* of enteroviruses [18].

## 4. Enteroviruses in Oncolytic Virotherapy

Oncolytic virotherapy refers to the use of replicating viruses to kill cancer cells. Viruses possess a tropism to different cell types due to specific virus-cell surface receptor interactions by which a virus only enters compatible target cells. Coincidentally, many viruses use receptors that are overexpressed in cancer cells and hence are capable of entering them. Replicating viruses often lyse the cancer cells, or alternatively mark them in the cell’s immune system. The first patient trials with oncolytic viruses were already done in the 1950s [56,57], and since then many viruses including adenovirus, vaccinia virus, Newcastle disease virus, herpes simplex virus, and several enteroviruses (poliovirus, coxsackieviruses and echoviruses) have been studied for their antitumoral activity. Despite encouraging results in animal models, there are currently only two accepted oncolytic virotherapy products on the market; Oncorine (a chimeric type 2/5 adenovirus with a partial deletion in the E1B gene) has been approved in China as a therapy against head and neck cancer [58]. Very recently, the U.S. Food and Drug Administration (FDA) approved talimogene laherparepvec (T-VEC), which is an engineered herpes simplex virus, to treat advanced melanoma. In addition, there is an enterovirus preparation called RIGVIR, which (presumably) is approved for clinical use in Latvia, and is also sold to other countries, but the molecular and clinical data (in English) on this virus “drug” is limited. Recently, RIGVIR was shown to prolong survival in early-stage melanoma patients [59]. Two oncolytic enteroviruses, poliovirus and coxsackievirus A21, have entered clinical phase trials.

Enteroviruses are promising tools in cancer therapy due to their ability to bind to specific cell surface receptors that are overexpressed in cancer cells. They have been used to eradicate cancer cells and tumors from both cell cultures and xenograft mouse models. Enteroviruses can be either native, cell-adapted viruses or genetically modified with improved properties such as improved cell tropism, reduced pathogenicity, increased ability to replicate and ability to express cytotoxic proteins in cancer cells [23,31]. However, before enteroviruses can be used in cancer therapies they have to have certain attributes that ensure patient safety: They should only infect cancer cells while leaving normal cells unaffected, and they should not cause severe illness or side effects. They should also be suitable for repeated treatments while allowing eradication by the host immune system. Studies on the oncolytic poliovirus vector have suggested that parameters defining the optimal oncolytic vector can rarely be attributed to single factors such as those explaining virus entry into specific cells via a specific receptor, or virus replication in specific cell types. Instead, there seems to be complicated interplay between extracellular factors, e.g., receptor expression and/or tissue type–specific receptor function, and intracellular factors, e.g., the protein synthesis machinery and its regulation in malignancy [60]. Thus, further studies are needed to identify the factors that contribute to the use of any enterovirus in virotherapy.

### 4.1. Poliovirus (PV)

As indicated earlier in this paper, the poliovirus vector has been extensively studied in the past 30 years for use in gene therapy. The natural neurotropism of poliovirus makes the PV vector the prime candidate for treatment of primary tumors originating from the CNS, although it may also be effective against other types of cancer including breast cancer and bone and soft tissue sarcomas [61]. Poliovirus infection is dependent on a single receptor, Nectin-like molecule 5 (Necl-5), which is highly expressed in malignant glioma cells [62,63], thereby potentiating poliovirus infectivity in them. However, expression of Necl-5 also mediates infectivity to lower motor neurons, causing serious neuropathogenicity. To overcome this, neuronal replication of the prototype oncolytic poliovirus called PVS-RIPO was abolished by replacing the poliovirus internal ribosome entry site (IRES) with a human rhinovirus serotype 2 (RV-A2) IRES site. This modification of the PV vector eliminated its ability to cause poliomyelitis in mice transgenic for Necl-5 and in *Cynomolgus macaques*, but did not reduce the cytopathogenicity of the virus vector in malignant cell types that express Necl-5 [64,65,66]. The toxicity and antitumor activity of PVS-RIPO was further evaluated in rodent models of glioblastoma multiforme neoplastic meningitis by using intrathecal delivery. It was demonstrated that there was no clinical or histologic evidence of toxicity. In efficacy studies, median survival was increased by 174% from 8.5 days in the group of rats treated with ultraviolet light–inactivated virus to 15 days in the rats treated with PVS-RIPO. The results suggested that intrathecal treatment with PVS-RIPO may be useful for the treatment of neoplastic meningitis in patients with glioblastoma multiforme (GBM), providing a rationale for clinical trials [67].

PVS-RIPO has been shown to be a very stable virus, alleviating the biosafety concerns. The vector has been serially passaged in cell cultures as well as in xenografts and shown to be very stable [60,66]. Even though the virus vector that was recovered from intermediate lesions 10 days after administration was estimated to have gone through at least 15 rounds of replication, cellular analyses indicated that it retained tumor-specificity. Moreover, full-length genome sequencing indicated that PVS-RIPO remained genetically stable [31,60]. FDA granted investigational new drug (IND) status for PVS-RIPO in 2011, which prompted PVS-RIPO to enter phase I clinical trials against recurrent GBM. Results of a phase I trial evaluating PVS-RIPO delivered intratumorally by convection-enhanced delivery (CED) indicated that PVS-RIPO can be safely used with encouraging efficacy results. Expanded phase I studies with additional patients are on-going (ClinicalTrials.gov Identifier: NCT01491893).

From a mechanistic view, the oncolytic PV vector not only kills tumor cells immediately after the injection but also awakens inflammatory reaction, which sets up secondary immune responses. Toyoda *et al.* [68] studied the ability of a neuro-attenuated poliovirus termed A133Gmono-crePV to induce an immunological response against cancer cells. The aim was to provoke the release of tumor antigens that would recruit, activate and load antigen-presenting cells for presentation to the T cells. Transgenic CD155 tg A/J mice were transplanted with neuroblastoma cells (NB) followed by virus challenge. Intratumoral injections of the neuro-attenuated poliovirus induced a CD8^+^ T cell–mediated immunity against NB. Vaccination of naïve mice by poliovirus vector–infected neuroblastoma lysate was also able to induce a potent antitumor immunity. The study suggested that *in vitro* poliovirus infection of neuroblastoma cells turns the target cells into a potent tumor immunogen, and this strategy should therefore be considered when designing therapies against poorly immunogenic human tumors [69].

### 4.2. Coxsackievirus 21 (CV-A21)

Coxsackievirus 21 (CV-A21 Kuykendall strain) is one of the most studied oncolytic enteroviruses, and rather surprisingly, its properties are based on receptor tropism via natural cell adaptation. Taxonomically, CV-A21 belongs to the *Enterovirus C* species, which include viruses that are mildly pathogenic and cause mainly upper respiratory symptoms. CV-A21 uses ICAM-1 and DAF receptors to adhere and infect cells. Compared to normal cells, the expression of these receptors is much higher in cancer cells and in human tumors. Oncolytic properties of CV-A21 were first assessed against *in vitro* cell cultures and *in vivo* xenografts of malignant human melanoma cells [70]. *In vitro* studies established that human melanoma cells express elevated levels of ICAM-1/DAF receptors and are highly susceptible to CV-A21 infection. A single-dose administration of CV-A21 resulted in a reduction of tumor size and growth in severe combined immunodeficiency (SCID) mice bearing multiple s.c. melanoma xenografts. Further studies demonstrated that the virus infects many malignant myeloma (MM) *in vitro* including U266, RPMI-8226 and NCI-H929 which express ICAM-1 and DAF receptors, but not PBMC (peripheral blood mononuclear) cells, which were used as a control. Similar effects were detected in *ex vivo* experiments with clinical bone marrow samples. CV-A21 has also been shown to be effective in breast cancer cell lines (BT-20, SK-BR-3, ZR-75-1, MDA-MB-231, -453, -157 and -361, MCF-7 and T-47D) [71]. A significant result from the breast cancer study was that intravenous injection of CV-A21 resulted in considerable reduction of the size of the primary tumors and eradication the metastasized tumors in SCID mice. This indicated that CV-A21 has tropism to cancer cells in a multicellular environment.

The most recent studies on oncolytic CV-A21 have been performed using a bioselected/cell-adapted variant of CV-A21, CV-A21-DAFv, which was generated by serial passage of CV-A21 on DAF-expressing, ICAM-1–negative rhabdomyosarcoma (RD) cells [72]. Comparison of parental CV-A21 with CV-A21-DAFv revealed that the latter was capable of lysing prostate cancer cell lines, and it possessed more efficient oncolytic properties, requiring a lower viral dose to achieve lytic destruction [73]. CV-A21-DAFv has been shown to bind to only the DAF receptor, which may explain its enhanced tropism to cancer cells. The oncolytic CV-A21, which is being evaluated in phase I and II clinical trials, is called CAVATAK™, and currently there are four completed clinical studies, one active study and two in the recruiting stage. Thus, CAVATAK™ represents a fine example of an oncolytic enterovirus that has not been genetically modified but adapted to use a specific cell surface receptor to enter and destroy cancer cells.

CV-A21 has also been used in combination with doxorubicin hydrochloride, which is a widely used anti-cancer chemotherapy drug. Flow cytometry analysis demonstrated that the human breast, colorectal, and pancreatic cancer cell lines examined expressed moderate levels of surface ICAM-1 and DAF, while a normal breast cell line expressed only minimal levels. When CV-A21 was combined with doxorubicin hydrochloride, synergistically enhanced cell death was observed when CV-A21 was administered both simultaneously or 24 h prior to doxorubicin hydrochloride exposure. Doxorubicin hydrochloride had no effect on CV-A21 replication. Through the use of an orthotopic (MDA-MB-231-luc) xenograft SCID mouse model of human breast cancer, it was also shown that a single intravenous injection of CV-A21 in combination with an intraperitoneal injection of doxorubicin hydrochloride resulted in significantly greater tumor reduction compared to either agent alone. This study demonstrated that CV-A21 in combination with doxorubicin hydrochloride effectively and potently targets human breast, colorectal and pancreatic cancer cell lines *in vitro* and *in vivo*, and offers a new therapeutic regimen for cancer therapy [74].

Instead of using virus vector preparations, the ability of CV-A21 to initiate oncolytic virus infection has been tested using a non-viral vector formulation. Plasmid-bearing CV-A21 genome cloned under the control of the T7 promoter was *in vitro* transcribed, and viral RNA was injected into KAS6/1 myeloma xenografts. High titers of infectious CV-A21 virions were detected in the bloodstream 48 h post-injection, which resulted in rapid tumor regression. Dose-response studies showed that an effective oncolytic infection could be established both by intravenous and intratumoral injection of infectious RNA. Treatment outcomes were comparable between intratumoral injection of naked RNA or infectious CV-A21 virus particles [75].

### 4.3. Other Enteroviruses

Besides poliovirus and CV-A21, there are number of experiments in which oncolytic properties of selected enteroviruses have been used. Miyamoto *et al.* [76] designed a massive two-step screening experiment to identify potential oncolytic enterovirus candidates. Screening was carried out in different human cancer cell lines including non–small cell lung cancer (NSCLC; A549, LK-87), colon cancer (Caco-2, DLD-1), pancreatic cancer (AsPC-1, PANC-1), renal cancer (A-498 and Caki-1), breast cancer (MCF7), cervical cancer (HeLa S3), rhabdomyosarcoma (RD), T-cell leukemia (HuT 102) and the HS-5 bone marrow stroma cell line. Out of 28 enteroviruses tested (including PV, CV-A21 and many echoviruses), they surprisingly found that CV-B2 (Ohio-1 strain), CV-B3 (Nancy strain) and CV-B4 (JVB strain) were the most effective and were selected for the second step because of their exclusive oncolytic effects in the A549 and LK-87 cells. In the second step these viruses were tested in other NSCLC cell lines (H1299, H460, PC-9, EBC-1, LK-2 and Sq-1), in two lung squamous cell carcinoma cell lines (QG-56 and QG-95) and in two normal lung cell lines (lung fibroblast NHLF and MRC-5). CV-B3 was the only virus that induced marked cytolysis in all cancer cell lines but not in lung fibroblast cell lines. However, *in vivo* studies in BALB/c mice indicated that even if suppression of A549- and EBC-1–induced tumor growth was observed and anti-tumor immune cells were awakened, virus infection also caused systemic disease symptoms. This calls for genetic modification of CV-B3 properties, meaning the attenuation of virus cytotoxic effects in normal tissues while retaining oncolytic properties.

Antitumoral properties of several echoviruses have been studied because they often cause subclinical infection while having a capacity to infect cancer cells specifically. Echovirus 1 (E-1) is the only enterovirus that uses integrin α2β1, a receptor which is abundantly expressed in cancer cell lines, for cellular entry. Shafren *et al.* [77] studied oncolytic properties against eight ovarian cancer cell lines, and demonstrated that E-1 was able to infect and destroy all cancer cell types. The oncolytic effect caused by E-1 was found to be stronger than that caused by CV-A21 or echovirus 7 (EV7). They also suggested that because E-1 binds to the integrin α2β1 receptor, which is also used by collagen 1, it establishes a competitive situation, which results in the prevention of cancer cell metastasis. E-1 infection also destroyed the cancer cells in three-dimensional (3D) cancer cell experiments. In the SCID mouse, injection of E-1 directly into the cancer cell transplantation or further from it produced a significant reduction in the number of cancer cells. However, the virus was cleared within seven days, which may have implications for repeated use. In another study, E-1 was found to be effective in stomach cancer cell lines (MKN-45, AGS, Hs746T and NCI-N87 [78]. E-1 infected three of the four cancer cell lines and tumor size in SCID mice was reduced considerably in size after a single dose of the virus.

Israelsson *et al.* [79] also investigated the oncolytical potential of echoviruses (E-12 Travis, E-15 Charleston, E-17 CHHE-29, E-26 Coronel and E-29 JV-10) in six colon cancer cell lines (CaCo-2, HT29, LoVo, SW480, SW620 and T84). The infectivity of echoviruses in SW480 and SW620 cell lines was found to be interesting because they originate from the same patient. SW480 was established from a primary adenocarcinoma of the colon while SW620 was established from a lymph node metastasis one year later. Results showed that echoviruses induced cytolysis faster in SW480 than in SW620. The authors suggested that there may be a “sliding therapeutic window” during cancer progression when the oncolytic virus is most effective. The conclusion of the study was that echoviruses 12, 17 and 26 show the greatest promise in potential oncolytic virotherapy against colon cancer.

Similarly to CV-A21, other viruses in the *Enterovirus C* species use ICAM-1/DAF receptors to adhere and infect cells. Oncolytic properties of the CV-A13 Flores strain, CV-A15 G-9 strain and CV-A18 G-13 strain have been studied in melanoma cell lines and in SCID mice and compared to CV-A21 [80]. Although CV-A21 infected melanoma cell lines more efficiently, the other viruses were used in the SCID mouse model; CV-A18 was shown to eradicate tumors in 48 days in five out of five of the cases while the ratios for CV-A15 and CV-A13 were two of five and zero of five tumors, respectively. Interestingly, these viruses are taxonomically related, and currently CV-A15 has been reclassified as a strain of CV-A11 while CV-A18 belongs to the CV-A13 serotype. This suggests that subtle differences within genomic sequences of the same type define the oncolytic properties.

## 5. Future Perspectives

Recent advances in enterovirus vector development suggest that virus vectors with different properties can be generated in a matter of a few weeks. The progress in NGS sequencing of enteroviruses also allows determination of the exact 5′- and 3′-end terminal sequences, which allows precise cloning of viral genomes directly into vector form. Combined with gene synthesis possibilities, novel vectors with desired sequence modifications are readily available. However, the compact virus structure, insert size and site of insertion still set limits to modifications. Enteroviruses use a polyprotein expression strategy, and any modifications may be reverted to the native virus genome within a matter of a few rounds of cell passage. The maximum size of the insert depends on the vector while the vector (or background virus type) defines the usability. Further studies are thus needed for any virus vector types to elucidate their usefulness in vector use. Many enteroviruses possess tropism to cancer cells or specific cell types, or can be adapted to infect them. Alternatively, enteroviruses can potentially be modified to make them visible to the immune system, similarly to the T-VEC herpesvirus vector. Since many enteroviruses that have been tested in oncolytic virotherapy are only mildly pathogenic, they may provide safe(r) alternatives to other oncolytic viruses.

## Figures and Tables

**Table 1:** Summary of enterovirus vectors and cloning strategies/sites.

Virus Type (and Receptor)	Strain	Insert	Site of Insertion	Reference
Poliovirus 1; PV-1 (CD155/Neclin-5)	Mahoney	BDNF with 3C^pr^°	Between 5′ UTR and VP4 gene	[34]
PV-1	Attenuated Sabin	RV-A2 IRES site with either E. coli FimH antigen, HIV Tat, SIV p17 or eGFP gene flanked by 2A^pr^°	Replacing poliovirus’s IRES region	[35]
PV-1	Mahoney	MIL-10 flanked by FMDV self-cleavage site and 2A^pr^°	Replacing VP3 and VP1 genes	[36]
PV-1	Mahoney	Helicobacter pyroli UreB gene	Replacing VP2-, VP3 and VP1 genes	[37]
PV-1	Mahoney	HPV-16 L1 shell protein as a whole (1600 nt) or partially (about 270 nt) flanked by 2A^pr^°	Between VP1 and 2A genes	[38]
Coxsackievirus B3; CV-B3 (CAR)	Attenuated CV-B3/0	MIL-4 flanked by 2A^pr^°	Between VP1 ja 2A^pr^° genes	[7]
CV-B3	CV-B3/0	HAdV-2′s L1 hexon antigen	Between VP1 and 2A^pr^° genes	[39]
CV-B3	CV-B3 H3	GFP	Between 5′ UTR region and VP4 gene	[40]
CV-B3	CV-B3 H3	GFP	Between 5′ UTR and VP4 gene	[9]
Coxsackievirus B4; CV-B4 (CAR)	Attenuated CV-B4 JVB	Ovalbumin gene	DE loop of the VP1 gene	[28]
CV-B4	Attenuated CV-B4 JVB	HIV-1 p24^gag^ gene fragments	Between 5′ UTR region and VP4 gene and within VP1 gene	[41]
Coxsackievirus A9; CV-A9 (αVβ3, αVβ6, β2M)	Griggs	eGFP	Between VP1 and 2A gene	[18]

Abbreviations: BDNF; brain-derived neurotropic factor, CAR; Coxsackie-adenovirus receptor; CV-B3, CV-B4, CV-A9; coxsackievirus B3, B4, A9, (e)GFP; (enhanced) green fluorescent protein, HAdV-2; human adenovirus type 2, HIV-1; human immunodeficiency virus 1, HPV; human papilloma virus, HRV; human rhinovirus, IRES; internal ribosome entry site, MCS; multiple cloning site, MIL-10, MIL-4; mouse interleukin 10 and 4, PV-1; poliovirus 1, SIV; simian immunodeficiency virus, UTR; untranslated region.

## References

[1] Tuthill T.J., Groppelli E., Hogle J.M., Rowlands D.J. (2010). Picornaviruses. Curr. Top. Microbiol. Immunol..

[2] Hulo C., de Castro E., Masson P., Bougueleret L., Bairoch A., Xenarios I., le Mercier P. (2011). ViralZone: A knowledge resource to understand virus diversity. Nucleic Acids Res..

[3] Whitton J.L., Cornell C.T., Feuer R. (2005). Host and virus determinants of picornavirus pathogenesis and tropism. Nat. Rev. Microbiol..

[4] Van der Linden L., Wolthers K.C., van Kuppeveld F.J. (2015). Replication and Inhibitors of Enteroviruses and Parechoviruses. Viruses.

[5] Rossmann M.G., He Y., Kuhn R.J. (2002). Picornavirus-receptor interactions. Trends Microbiol..

[6] Merilahti P., Koskinen S., Heikkila O., Karelehto E., Susi P. (2012). Endocytosis of integrin-binding human picornaviruses. Adv. Virol..

[7] Chapman N.M., Kim K.S., Tracy S., Jackson J., Hofling K., Leser J.S., Malone J., Kolbeck P. (2000). Coxsackievirus expression of the murine secretory protein interleukin-4 induces increased synthesis of immunoglobulin G1 in mice. J. Virol..

[8] Hughes P.J., Horsnell C., Hyypia T., Stanway G. (1995). The coxsackievirus A9 RGD motif is not essential for virus viability. J. Virol..

[9] Lim B.K., Shin J.O., Lee S.C., Kim D.K., Choi D.J., Choe S.C., Knowlton K.U., Jeon E.S. (2005). Long-term cardiac gene expression using a coxsackieviral vector. J. Mol. Cell. Cardiol..

[10] Slifka M.K., Pagarigan R., Mena I., Feuer R., Whitton J.L. (2001). Using recombinant coxsackievirus B3 to evaluate the induction and protective efficacy of CD8^+^ T cells during picornavirus infection. J. Virol..

[11] Meyer R.G., Meyer-Ficca M.L., Kaiser H., Selinka H.C., Kandolf R., Kupper J.H. (2004). Plasmid-based generation of recombinant coxsackievirus B3 particles carrying capsid gene replacement replicons. Virus Res..

[12] Gullberg M., Tolf C., Jonsson N., Polacek C., Precechtelova J., Badurova M., Sojka M., Mohlin C., Israelsson S., Johansson K. (2010). A single coxsackievirus B2 capsid residue controls cytolysis and apoptosis in rhabdomyosarcoma cells. J. Virol..

[13] Israelsson S., Gullberg M., Jonsson N., Roivainen M., Edman K., Lindberg A.M. (2010). Studies of Echovirus 5 interactions with the cell surface: Heparan sulfate mediates attachment to the host cell. Virus Res..

[14] Gow J.W., McGill M.M., Behan W.M., Behan P.O. (1996). Long RT-PCR amplification of full-length enterovirus genome. BioTechniques.

[15] Leister D., Thompson R. (1996). Production of full-length cDNA from a picornaviral genome by RT-PCR. Trends Genet..

[16] Tellier R., Bukh J., Emerson S.U., Purcell R.H. (1996). Amplification of the full-length hepatitis A virus genome by long reverse transcription-PCR and transcription of infectious RNA directly from the amplicon. Proc. Natl. Acad. Sci. USA.

[17] Tellier R., Bukh J., Emerson S.U., Miller R.H., Purcell R.H. (1996). Long PCR and its application to hepatitis viruses: Amplification of hepatitis A, hepatitis B, and hepatitis C virus genomes. J. Clin. Microbiol..

[18] Heikkila O., Kainulainen M., Susi P. (2011). A combined method for rescue of modified enteroviruses by mutagenic primers, long PCR and T7 RNA polymerase-driven *in vivo* transcription. J. Virol. Methods.

[19] Boot H.J., Schepp R.M., van Nunen F.J., Kimman T.G. (2004). Rapid RT-PCR amplification of full-length poliovirus genomes allows rapid discrimination between wild-type and recombinant vaccine-derived polioviruses. J. Virol. Methods.

[20] Poirier J.T., Reddy P.S., Idamakanti N., Li S.S., Stump K.L., Burroughs K.D., Hallenbeck P.L., Rudin C.M. (2012). Characterization of a full-length infectious cDNA clone and a GFP reporter derivative of the oncolytic picornavirus SVV-001. J. Gen. Virol..

[21] Tan le V., Tuyen N.T., Thanh T.T., Ngan T.T., van H.M., Sabanathan S., van T.T., Thanh le T.M., Nguyet L.A., Geoghegan J.L. (2015). A generic assay for whole-genome amplification and deep sequencing of enterovirus A71. J. Virol. Methods.

[22] Herold J., Andino R. (2000). Poliovirus requires a precise 5′ end for efficient positive-strand RNA synthesis. J. Virol..

[23] Israelsson S., Savneby A., Ekstrom J.O., Jonsson N., Edman K., Lindberg A.M. (2014). Improved replication efficiency of echovirus 5 after transfection of colon cancer cells using an authentic 5′ RNA genome end methodology. Investig. New Drugs.

[24] Pelletier J., Sonenberg N. (1988). Internal initiation of translation of eukaryotic mRNA directed by a sequence derived from poliovirus RNA. Nature.

[25] Kitamura N., Semler B.L., Rothberg P.G., Larsen G.R., Adler C.J., Dorner A.J., Emini E.A., Hanecak R., Lee J.J., van der Werf S. (1981). Primary structure, gene organization and polypeptide expression of poliovirus RNA. Nature.

[26] Arnold G.F., Resnick D.A., Smith A.D., Geisler S.C., Holmes A.K., Arnold E. (1996). Chimeric rhinoviruses as tools for vaccine development and characterization of protein epitopes. Intervirology..

[27] Burke K.L., Evans D.J., Jenkins O., Meredith J., D’Souza E.D., Almond J.W. (1989). A cassette vector for the construction of antigen chimaeras of poliovirus. J. Gen. Virol..

[28] Halim S.S., Ostrowski S.E., Lee W.T., Ramsingh A.I. (2000). Immunogenicity of a foreign peptide expressed within a capsid protein of an attenuated coxsackievirus. Vaccine.

[29] Evans D.J., McKeating J., Meredith J.M., Burke K.L., Katrak K., John A., Ferguson M., Minor P.D., Weiss R.A., Almond J.W. (1989). An engineered poliovirus chimaera elicits broadly reactive HIV-1 neutralizing antibodies. Nature.

[30] Crotty S., Miller C.J., Lohman B.L., Neagu M.R., Compton L., Lu D., Lu F.X., Fritts L., Lifson J.D., Andino R. (2001). Protection against simian immunodeficiency virus vaginal challenge by using Sabin poliovirus vectors. J. Virol..

[31] Dobrikova E.Y., Broadt T., Poiley-Nelson J., Yang X., Soman G., Giardina S., Harris R., Gromeier M. (2008). Recombinant oncolytic poliovirus eliminates glioma *in vivo* without genetic adaptation to a pathogenic phenotype. Mol. Ther..

[32] Cho S.P., Lee B., Min M.K. (2000). Recombinant polioviruses expressing hepatitis B virus-specific cytotoxic T-lymphocyte epitopes. Vaccine.

[33] Kim D.S., Cho Y.J., Kim B.G., Lee S.H., Nam J.H. (2010). Systematic analysis of attenuated Coxsackievirus expressing a foreign gene as a viral vaccine vector. Vaccine.

[34] Jia Q., Liang F., Ohka S., Nomoto A., Hashikawa T. (2002). Expression of brain-derived neurotrophic factor in the central nervous system of mice using a poliovirus-based vector. J. Neurovirol..

[35] Dobrikova E.Y., Florez P., Gromeier M. (2003). Structural determinants of insert retention of poliovirus expression vectors with recombinant IRES elements. Virology.

[36] Jackson C.A., Messinger J., Peduzzi J.D., Ansardi D.C., Morrow C.D. (2005). Enhanced functional recovery from spinal cord injury following intrathecal or intramuscular administration of poliovirus replicons encoding IL-10. Virology.

[37] Smythies L.E., Novak M.J., Waites K.B., Lindsey J.R., Morrow C.D., Smith P.D. (2005). Poliovirus replicons encoding the B subunit of Helicobacter pylori urease protect mice against H. pylori infection. Vaccine.

[38] Van Kuppeveld F.J., de Jong A., Dijkman H.B., Andino R., Melchers W.J. (2002). Studies towards the potential of poliovirus as a vector for the expression of HPV 16 virus-like-particles. FEMS Immunol. Med. Microbiol..

[39] Hofling K., Tracy S., Chapman N., Kim K.S., Smith Leser J. (2000). Expression of an antigenic adenovirus epitope in a group B coxsackievirus. J. Virol..

[40] Feuer R., Mena I., Pagarigan R., Slifka M.K., Whitton J.L. (2002). Cell cycle status affects coxsackievirus replication, persistence, and reactivation *in vitro*. J. Virol..

[41] Halim S.S., Collins D.N., Ramsingh A.I. (2000). A therapeutic HIV vaccine using coxsackie-HIV recombinants: A possible new strategy. AIDS Res. Hum. Retroviruses..

[42] Alexander L., Lu H.H., Gromeier M., Wimmer E. (1994). Dicistronic polioviruses as expression vectors for foreign genes. AIDS Res. Hum. Retroviruses..

[43] Choi W.S., Pal-Ghosh R., Morrow C.D. (1991). Expression of human immunodeficiency virus type 1 (HIV-1) gag, pol, and env proteins from chimeric HIV-1-poliovirus minireplicons. J. Virol..

[44] Dufresne A.T., Dobrikova E.Y., Schmidt S., Gromeier M. (2002). Genetically stable picornavirus expression vectors with recombinant internal ribosomal entry sites. J. Virol..

[45] Yim T.J., Tang S., Andino R. (1996). Poliovirus recombinants expressing hepatitis B virus antigens elicited a humoral immune response in susceptible mice. Virology.

[46] Tang S., van Rij R., Silvera D., Andino R. (1997). Toward a poliovirus-based simian immunodeficiency virus vaccine: Correlation between genetic stability and immunogenicity. J. Virol..

[47] Bledsoe A.W., Gillespie G.Y., Morrow C.D. (2000). Targeted foreign gene expression in spinal cord neurons using poliovirus replicons. J. Neurovirol..

[48] Kim D.S., Nam J.H. (2010). Characterization of attenuated coxsackievirus B3 strains and prospects of their application as live-attenuated vaccines. Expert Opin. Biol. Ther..

[49] Reimann B.Y., Zell R., Kandolf R. (1991). Mapping of a neutralizing antigenic site of Coxsackievirus B4 by construction of an antigen chimera. J. Virol..

[50] Miller J.P., Geng Y., Ng H.L., Yang O.O., Krogstad P. (2009). Packaging limits and stability of HIV-1 sequences in a coxsackievirus B vector. Vaccine.

[51] Yin H., Berg A.K., Tuvemo T., Frisk G. (2002). Enterovirus RNA is found in peripheral blood mononuclear cells in a majority of type 1 diabetic children at onset. Diabetes.

[52] Moya-Suri V., Schlosser M., Zimmermann K., Rjasanowski I., Gurtler L., Mentel R. (2005). Enterovirus RNA sequences in sera of schoolchildren in the general population and their association with type 1-diabetes-associated autoantibodies. J. Med. Microbiol..

[53] Roivainen M., Piirainen L., Hovi T., Virtanen I., Riikonen T., Heino J., Hyypia T. (1994). Entry of coxsackievirus A9 into host cells: Specific interactions with alpha v beta 3 integrin, the vitronectin receptor. Virology.

[54] Williams C.H., Kajander T., Hyypia T., Jackson T., Sheppard D., Stanway G. (2004). Integrin α_v_β_6_ is an RGD-dependent receptor for coxsackievirus A9. J. Virol..

[55] Liu Z., Wang F., Chen X. (2008). Integrin α_v_β_3_ Targeted Cancer Therapy. Drug Dev. Res..

[56] Dey M., Auffinger B., Lesniak M.S., Ahmed A.U. (2013). Antiglioma oncolytic virotherapy: Unattainable goal or a success story in the making?. Future Virol..

[57] Kelly E., Russell S.J. (2007). History of oncolytic viruses: Genesis to genetic engineering. Mol. Ther..

[58] Ma G., Shimada H., Hiroshima K., Tada Y., Suzuki N., Tagawa M. (2009). Gene medicine for cancer treatment: Commercially available medicine and accumulated clinical data in China. Drug Des. Devel.Ther..

[59] Donina S., Strele I., Proboka G., Auzins J., Alberts P., Jonsson B., Venskus D., Muceniece A. (2015). Adapted ECHO-7 virus Rigvir immunotherapy (oncolytic virotherapy) prolongs survival in melanoma patients after surgical excision of the tumour in a retrospective study. Melanoma Res..

[60] Goetz C., Gromeier M. (2010). Preparing an oncolytic poliovirus recombinant for clinical application against glioblastoma multiforme. Cytokine Growth Factor Rev..

[61] Atsumi S., Matsumine A., Toyoda H., Niimi R., Iino T., Nakamura T., Matsubara T., Asanuma K., Komada Y., Uchida A., Sudo A. (2012). Oncolytic virotherapy for human bone and soft tissue sarcomas using live attenuated poliovirus. Int. J. Oncol..

[62] Merrill M.K., Bernhardt G., Sampson J.H., Wikstrand C.J., Bigner D.D., Gromeier M. (2004). Poliovirus receptor CD155-targeted oncolysis of glioma. Neuro. Oncol..

[63] Ochiai H., Moore S.A., Archer G.E., Okamura T., Chewning T.A., Marks J.R., Sampson J.H., Gromeier M. (2004). Treatment of intracerebral neoplasia and neoplastic meningitis with regional delivery of oncolytic recombinant poliovirus. Clin. Cancer. Res..

[64] Gromeier M., Alexander L., Wimmer E. (1996). Internal ribosomal entry site substitution eliminates neurovirulence in intergeneric poliovirus recombinants. Proc. Natl. Acad. Sci. USA.

[65] Gromeier M., Bossert B., Arita M., Nomoto A., Wimmer E. (1999). Dual stem loops within the poliovirus internal ribosomal entry site control neurovirulence. J. Virol..

[66] Gromeier M., Lachmann S., Rosenfeld M.R., Gutin P.H., Wimmer E. (2000). Intergeneric poliovirus recombinants for the treatment of malignant glioma. Proc. Natl. Acad. Sci. USA.

[67] Ochiai H., Campbell S.A., Archer G.E., Chewning T.A., Dragunsky E., Ivanov A., Gromeier M., Sampson J.H. (2006). Targeted therapy for glioblastoma multiforme neoplastic meningitis with intrathecal delivery of an oncolytic recombinant poliovirus. Clin. Cancer. Res..

[68] Toyoda H., Yin J., Mueller S., Wimmer E., Cello J. (2007). Oncolytic treatment and cure of neuroblastoma by a novel attenuated poliovirus in a novel poliovirus-susceptible animal model. Cancer Res..

[69] Toyoda H., Wimmer E., Cello J. (2011). Oncolytic poliovirus therapy and immunization with poliovirus-infected cell lysate induces potent antitumor immunity against neuroblastoma *in vivo*. Int. J. Oncol..

[70] Shafren D.R., Au G.G., Nguyen T., Newcombe N.G., Haley E.S., Beagley L., Johansson E.S., Hersey P., Barry R.D. (2004). Systemic therapy of malignant human melanoma tumors by a common cold-producing enterovirus, coxsackievirus a21. Clin. Cancer. Res..

[71] Skelding K.A., Barry R.D., Shafren D.R. (2009). Systemic targeting of metastatic human breast tumor xenografts by Coxsackievirus A21. Breast Cancer Res. Treat..

[72] Johansson E.S., Xing L., Cheng R.H., Shafren D.R. (2004). Enhanced cellular receptor usage by a bioselected variant of coxsackievirus a21. J. Virol..

[73] Berry L.J., Au G.G., Barry R.D., Shafren D.R. (2008). Potent oncolytic activity of human enteroviruses against human prostate cancer. Prostate.

[74] Skelding K.A., Barry R.D., Shafren D.R. (2012). Enhanced oncolysis mediated by Coxsackievirus A21 in combination with doxorubicin hydrochloride. Invest. New. Drugs.

[75] Hadac E.M., Kelly E.J., Russell S.J. (2011). Myeloma xenograft destruction by a nonviral vector delivering oncolytic infectious nucleic acid. Mol. Ther..

[76] Miyamoto S., Inoue H., Nakamura T., Yamada M., Sakamoto C., Urata Y., Okazaki T., Marumoto T., Takahashi A., Takayama K., Nakanishi Y., Shimizu H., Tani K. (2012). Coxsackievirus B3 is an oncolytic virus with immunostimulatory properties that is active against lung adenocarcinoma. Cancer Res..

[77] Shafren D.R., Sylvester D., Johansson E.S., Campbell I.G., Barry R.D. (2005). Oncolysis of human ovarian cancers by echovirus type 1. Int. J. Cancer..

[78] Haley E.S., Au G.G., Carlton B.R., Barry R.D., Shafren D.R. (2009). Regional administration of oncolytic Echovirus 1 as a novel therapy for the peritoneal dissemination of gastric cancer. J. Mol. Med..

[79] Israelsson S., Jonsson N., Gullberg M., Lindberg A.M. (2011). Cytolytic replication of echoviruses in colon cancer cell lines. Virol. J..

[80] Au G.G., Beagley L.G., Haley E.S., Barry R.D., Shafren D.R. (2011). Oncolysis of malignant human melanoma tumors by Coxsackieviruses A13, A15 and A18. Virol. J..

